# Role and Mechanism of PKC-δ for Cardiovascular Disease: Current Status and Perspective

**DOI:** 10.3389/fcvm.2022.816369

**Published:** 2022-02-15

**Authors:** Li-na Miao, Deng Pan, Junhe Shi, Jian-peng Du, Peng-fei Chen, Jie Gao, Yanqiao Yu, Da-Zhuo Shi, Ming Guo

**Affiliations:** ^1^Xiyuan Hospital, China Academy of Chinese Medical Sciences, Beijing, China; ^2^Department of Graduate School, Beijing University of Chinese Medicine, Beijing, China; ^3^China Heart Institute of Chinese Medicine, China Academy of Chinese Medical Sciences, Beijing, China

**Keywords:** cardiovascular disease, PKC-δ, kinase, mechanism, PKC

## Abstract

Protein kinase C (PKC) is a protein kinase with important cellular functions. PKC-δ, a member of the novel PKC subfamily, has been well-documented over the years. Activation of PKC-δ plays an important regulatory role in myocardial ischemia/reperfusion (IRI) injury and myocardial fibrosis, and its activity and expression levels can regulate pathological cardiovascular diseases such as atherosclerosis, hypertension, cardiac hypertrophy, and heart failure. This article aims to review the structure and function of PKC-δ, summarize the current research regarding its activation mechanism and its role in cardiovascular disease, and provide novel insight into further research on the role of PKC-δ in cardiovascular diseases.

## Introduction

The term cardiovascular disease (CVD) includes many different diseases, such as coronary heart disease, hypertension, heart failure, and atherosclerosis (1). The global prevalence of CVD increased from 271 million in 1990 to 523 million in 2019, and the corresponding mortality rates were 12.1 million in 1990 and approximately 18.6 million in 2019 (2). Although the treatment of cardiovascular diseases has greatly improved in recent years, these diseases still exhibit high morbidity and mortality. There is still a lack of research exploring new targets for the prevention and treatment of CVD. In recent years, studies have found that protein kinase C (PKC) plays a multifaceted role in the pathophysiology of heart development and many cardiovascular diseases.

Protein kinase C is a member of the AGC protein kinase superfamily. To date, 10 PKC family members have been found in mammalian tissues. These family members can be divided into three categories according to their dependency on lipid second messengers and calcium and ion coactivators for activation: conventional PKCs (α, β1, β2, γ), novel PKCs (δ, ε, η, θ), and atypical PKCs (ζ, λ/ι) (3– 5). The activation of conventional PKCs requires diglyceride (DAG), phosphatidylserine (PS), and Ca^2+^. Novel PKCs are regulated by DAG and PS but do not require Ca^2+^ for activation, and the activity of atypical PKCs is only stimulated by PS (6–8) (Table 1). Protein kinase C activation is a complicated process that includes membrane association of the enzymes, priming by phosphorylation, conformational changes induced by binding of proteins or second messengers (e.g., Ca^2+^, PS), and the release of a pseudo substrate. In addition, DAT and DAT-based phospholipids can also activate PKC (9–11). The activated PKC isoform protein plays a vital role in many important human diseases, including diabetes (PKC-β) (12), cancer (PKC-ε) (13), autoimmune diseases (PKC-θ) (14), Parkinson (PKC-δ) (15, 16) etc. PKC-α, PKC-β, PKC-ε, and PKC-δ are among the most studied molecules in CVD, participate in various CVD, such as atherosclerosis, hypertension, atrial fibrillation, and cardiac hypertrophy (17–22). Protein kinase C mediates CVD by regulating apoptosis in smooth muscle cells and cardiomyocytes, regulating endothelial function, and participating in the regulation of cardiac ion channels. PKC-α is involved in platelet activation in atherosclerosis, and inhibition of PKC-α can reduce thrombogenesis at atherosclerotic plaques (23). Both PKC-δ and PKC-ε are activated in the ischemic heart of humans and myocardial infarction (MI) model of animals. However, opposite effects were observed (21, 24, 25). Selective activation of PKC-ε protects mitochondrial function prior to ischemic events or during reperfusion by activating mitochondrial aldehyde dehydrogenase 2 (ALDH2) and removing toxic aldehyde-lipid peroxidation products (26). In addition, PKC-ε inhibits the activation of L-type calcium channels in cardiomyocytes and prevents fatal ventricular arrhythmias associated with ischemia-reperfusion (I/R) injury (27). In contrast, PKC-δ activation mediates damage mainly by activating mitochondrial pyruvate dehydrogenase kinase, which inhibits pyruvate dehydrogenase, ATP regeneration, and thus induces necrosis (28). Rats and mice with vascular restenosis are benefited by inhibition of PKCβ and PKC-δ therapy. PKC-δ inhibition and PKC-ε activation can also alleviate vascular disease after transplantation or interventional therapy (29). PKC-δ is one of the most important and studied targets of the cardiac G protein-coupled receptor signaling pathway. PKC-δ can mediate apoptosis, inflammation, vasoconstriction, and vasodilation of blood vessels, which plays a key role in the regulation of cardiac contractility, ischemic preconditioning, and cardiac structure remodeling. Activation of PKC-δ plays an important regulatory role in myocardial ischemia/reperfusion (IRI) injury and myocardial fibrosis, and is involved in many cardiovascular diseases, such as atherosclerosis, hypertension, myocardial hypertrophy, and heart failure. The role of conventional PKC in the pathogenesis of pathological CVD had already been systematically summarized (11, 30). This paper reviews the research progress of PKC-δ in the pathogenesis of pathological CVD to provide ideas for further study on the role of PKC-δ in the pathogenesis of cardiovascular disease.

**Table 1:** PKC isoform sub-family members.

**Type of PKC**	**Isoforms of PKC**	**Structural domain**	**Ligands**
Conventional PKCs	PKC-α PKC-β1 PKC-β2 PKC-γ	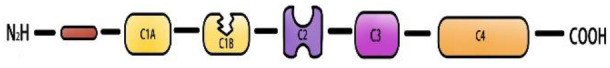	**C1B:** DAG / PS, **C2:** PIP_2_+Ca^2+^
Novel PKCs	PKC-δ PKC-ε PKC-η PKC-θ	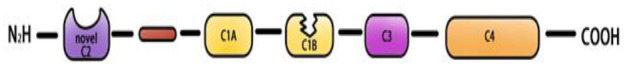	**C1:** DAG/PS
Atypical PKCs	PKC-ζ PKC-λ/ι	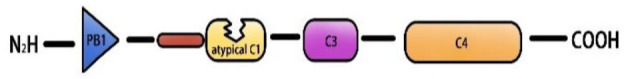	**C1:** PS

*DAG, diacylglycerol; PB1, Phox and Bem1; PIP2, phosphatidylinositol 4,5-bisphosphate; PS, phosphatidylserine*.

## Structure and Function of PKC-δ

### Structure of PKC-δ

**Figure 1:**
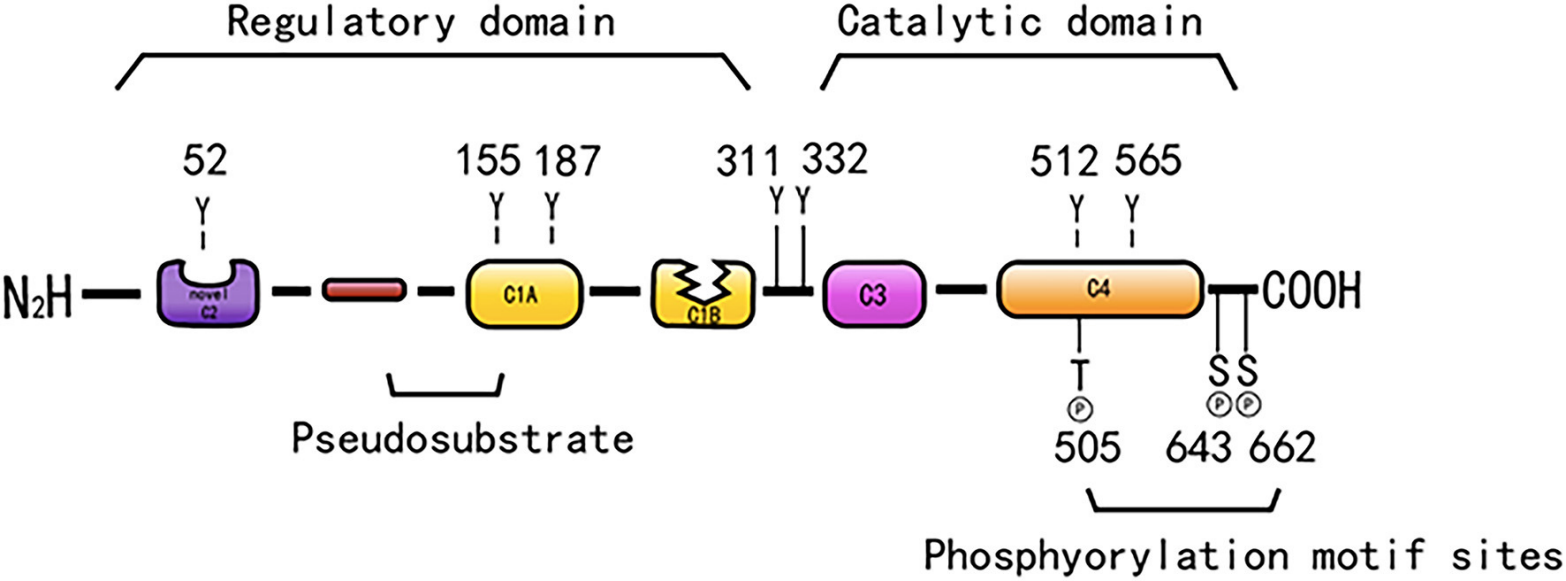
The structure of PKC-δ. Schematic showing pseudo substrate (red rectangle), C1 domain (yellow rectangle), C2 domain (purple rectangle), C3 domain (pink rectangle), C4 domain (orange rectangle). The cyan represents the connecting hinge segment, the kinase region, and the gray rectangle represents the C-terminal tail. In particular, w switch in C1B domain determines the affinity of DAG containing membrane.

Protein kinase C is a Ca^2+^ and lecithin-dependent protein kinase that is activated by Ca^2+^, acylglycerol (DAG), and phorbol ester (PS). PKC-δ is a member of the novel PKC family, which was cloned from a rat cDNA library in 1987 (31) and is widely present in various tissues, such as brain tissue and epithelial tissue cells, and its activation does not require Ca^2+^ involvement. Its molecular structure consists of a peptide chain, divided into four conserved regions (C1–C4) and five variable regions (V1–V5) (Figure 1). The C1 region of PKC-δ binds diacylglycerol and PS, which consists of a pseudosubstrate binding site and a zinc finger region, playing an essential role in its activation, and includes a tandem repeat of Cys-rich motifs named CIA and C1B (32). The C1A and C1B regions of PKC-δ are not equivalent, and the C1B region is the major PS-binding site of PKC-δ (33–35). PKC-δ lacks the authentic C2 region and instead has a C2-like region in the amino-terminal end of the molecule (36). The PKC-δ C2 domain differs from the C2 domain of traditional PKC, and it lacks key residues coordinating with Ca^2+^ (37). C3 is the ATP binding site. The C4 region contains a substrate-binding region that is required for the recognition of phosphorylated substrates. The C4 region harbors a phosphorylation motif site, Thr-505, in the activation loop, and the carboxyl-terminal end of the enzyme has two conserved phosphorylation sites, Ser-643, and Ser-662, which are hinge and hydrophobic motif sites, respectively (38).

PKC-δ consists of a regulatory region and a catalytic region that is separated by the V3 region. The regulatory region is composed of an NH2-terminal regulatory domain, which has a targeting effect that is related to the subcellular localization of the enzyme and regulates the activity of the enzyme. The catalytic region consists of a COOH-terminal catalytic domain, which can bind to a protein, a polypeptide substrate, and a phosphate donor ATP/GTP, and this domain can transfer a phosphate-based amino acid residue to the corresponding amino acid residue.

Both the catalytic and regulatory domains can be used to generate drugs that modulate PKC activity, leading to PKC inhibition or activation. The drug target regions of PKC-δ are mainly located at ATP-binding site in catalytic domain (39), DAG-binding site of C1 domain (40), and unique sequence of C2 domain (41). Generation of specific PKC subtype inhibitors is difficult due to high homolog of catalytic domain and conservation of ATP-binding site in PKC subtypes. In addition, it is also challenging to generate a drug that selectively binds to one of the isozymes C1 domains because of the high homolog of C1 domain and DAG-binding site. Drugs that target the C2 region and the inserted sequence (V region) between the domains show higher isozyme selectivity. Current drugs targeting PKC-δ include: Rottlerin (ATP-binding site), Bryostatin1 (C1 domain of PKC), and δV1-1 (Unique sequence of PKCδ -C2 domain) are the main drugs targeting PKC-δ.

In addition, PKC-δ has genetic polymorphisms, and conditional knockout mice can be generated through splicing of the PKC- δ gene, Prkcd. Conditional knockout mice are generated by regulating expression of exons (42). Conditional general PKC-δ knockout mice were generated by knockout of PKC-δ encoding gene (by deleting shared exons of PKC-δ I, II, IV, V, VI, and VII) through Cre-Lox recombination (42). In addition, conditional specific PKC-δ-suptype knockout mice were also reported. Leitges et al. generated PKC-δ II deletion mice by inserting a LacZ/neo cassette into the first transcribed exon of Prkcd (43). So far, nine subtypes of PKC-δ (I, II, III, IV, V, VI, VII, VIII, and IX) have been discovered (44–48). Among them, PKC-δ I, II, IV, V, VI, VII, and IX were found to be expressed in mice (44–46). PKC-δ I and III were found to be expressed in rats (47), whereas PKC-δ I and VIII were found to be expressed in human (48). And PKC-δ VIII is thought to be the human homolog of mouse PKC-δ II (48). In current studies, PKC-δ^−/−^ mice are mostly the knockout of PKC-δ I and II subtypes. Recently, Niino et al. generated a conditional knockout mice lack of PKC-δ I or II, IV, V, VI, and VII (42). Their study found that a wide range of PKC-δ subtypes knockout can inhibit fetal development and is associated with adult mice heart elastic fiber hyperplasia and lung inflammation (42).

## PKC-δ Activation Mechanisms

**Figure 2:**
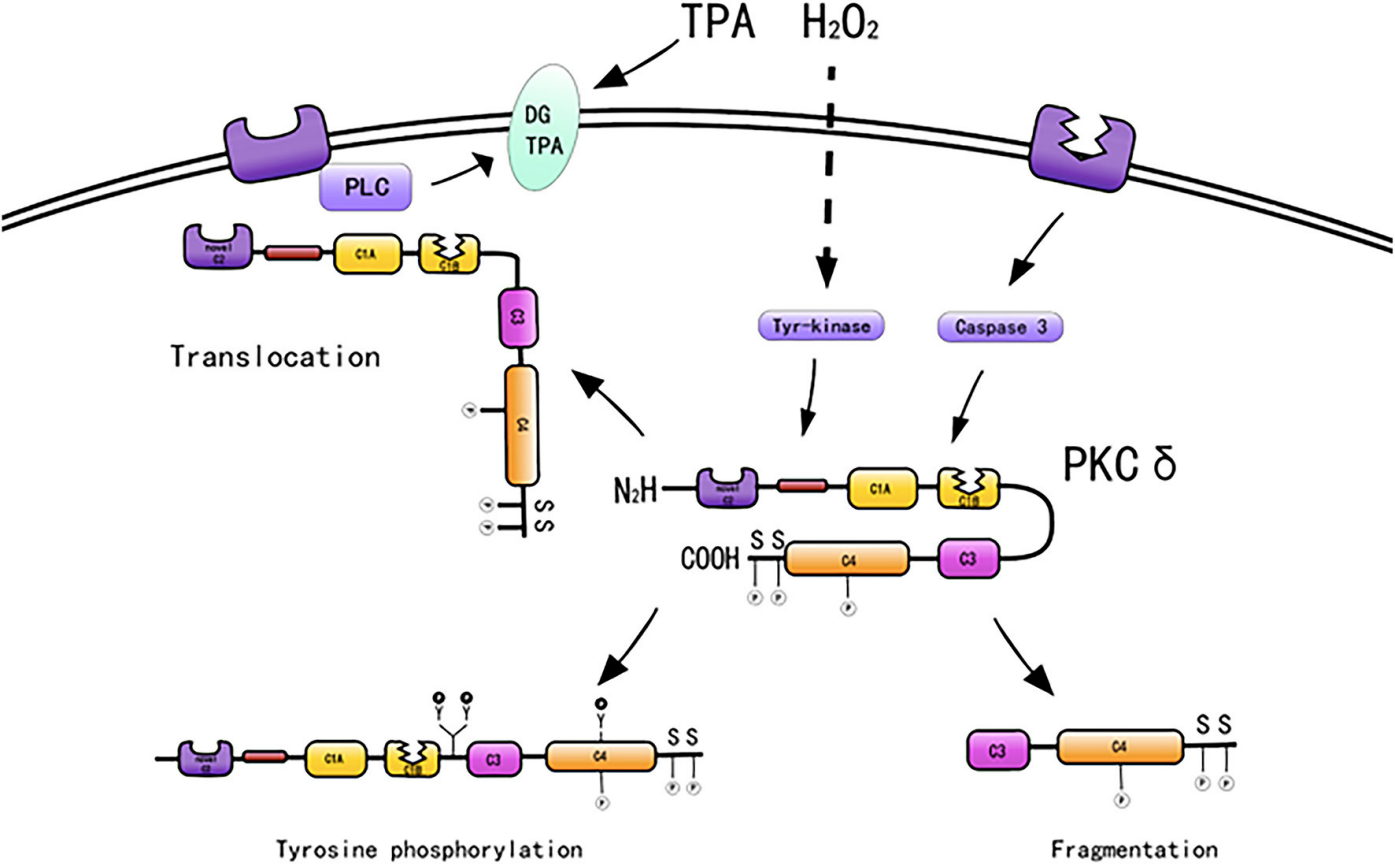
Activation mechanisms of PKC-δ [adapted from Ushio et al. (50)].

PKC-δ typically exists in the cytoplasm in an inactive form. Mechanisms regarding how PKC-δ is activated by various stimuli include binding of diacylglycerol and PS, phosphorylation at motif sites, tyrosine phosphorylation, membrane translocation, and proteolysis. In addition, stimulation by ATP and H_2_O_2_ also activates PKC-δ (49) (Figure 2).

### Diacylglycerol and Phorbol Ester

PKC-δ lacks a calcium-binding C2 domain and is maximally activated by DAG and PS in the absence of calcium (51). PKC-δ is activated by DAG, A variety of molecules, such as growth factors, PDGF, hormones, epidermal growth factors, and cytokines, when bound to their respective receptors, will activate members of the phospholipase C family, which will produce diacylglycerols, thereby activating PKC isoenzymes (52). PKC-δ serves as the receptor for tumor-promoting PSs, such as 12-O-tetradecanoylphorbol 13-acetate (TPA). 12-O-Tetradecanoylphorbol 13-acetate directly activates PKC-δ (53). Phorbol treatment induces PKC-δ overexpression and cell proliferation (54) and differentiation (55). For example, phorbol-treated NIH3T3 cells express PKC-δ and exhibit reduced cell growth (54), and TPA induces monocyte differentiation in 32D cells overexpressing PKC-δ (55).

### Phosphorylation at Motif Sites

Protein kinase C consists of a series of conserved phosphorylation sites, which are located in the kinase activating ring, the trans motif and the hydrophobic motif. Phosphorylation causes the kinase to mature and prolong its cellular half-life (11). PKC-δ has phosphorylation motif sites of conserved serine or threonine residues (5, 38). PKC-δ is phosphorylated in the hinge and hydrophobic motif sites in the carboxyl-terminal end region. PKC-δ phosphorylates cTnI at Ser23/Ser24 when activated by lipid cofactors (56). PKC-δ harbors an activation loop, hinge, and hydrophobic motif sites at Thr-505, Ser-643, and Ser-662, respectively, and these sites are substantially phosphorylated *in vivo* (57). The phosphorylation of PKC-δ at Thr505 is induced by PMA or the α1-adrenergic receptor agonist noradrenaline (norepinephrine) in cardiomyocytes (58). PKC-δ is phosphorylated *in vivo* by c-Fyn (59, 60), c-Src (61, 62), and growth factor receptor (59, 60).

### Tyrosine Phosphorylation

PKC-δ exhibits the most efficient tyrosine phosphorylation among the PKC family members, and different tyrosine residues are further phosphorylated upon cellular stimulation (57). Several tyrosine phosphorylation sites were found in the catalytic domain (tyr512 and tyr523), regulatory domain (tyr52, tyr155, and tyr187), and hinge region (tyr311 and tyr332) of PKC-δ. In contrast to phosphorylation at serine and threonine motif sites, the phosphorylated tyrosine residues identified to date, such as Tyr-311 and Tyr-332 of PKC-δ, are not conserved among members of the PKC family; however, Tyr-512 of PKC-δ represents an exception (57). PKC-δ is phosphorylated on tyrosine residues of cells stimulated by SRC or Ras transformation, H_2_O_2_, PMA, EGF, or PDGF. Frank et al. demonstrated the presence of distinct tyrosine kinase activation pathways of PKC-δ/PYK2/JAK2 utilized by H_2_O_2_ in vascular smooth muscle cells (VSMCs) (63). During oxidative stress, PKC-δ is activated by Src-mediated phosphorylation of an otherwise unutilized tyrosine residue in the enzyme (56, 64). A study indicated that tyrosine phosphorylation of PKC-δ may regulate its activity (59), and phosphorylation of tyrosine can both promote and inhibit the catalytic activity of PKC-δ. For example, the catalytic activity of PKC-δ was reduced by tyrosine phosphorylation in eRAS-transformed keratinocytes (60); however, PKC-δ phosphorylated at Tyr-311 *in vitro* shows enhanced catalytic activity (57). Tyrosine phosphorylation of PKC-δ modifies PKC-δ-dependent phosphorylation of cardiac troponin I (cTnI), a myofilament regulatory protein (56). In addition, tyrosine phosphorylation enhances the enzymatic activity in various cells stimulated by substances, such as PS, growth factors, and hormones (65–70). Many studies have relied on *in vitro* kinase analysis to address tyrosine phosphorylation-dependent changes in PKC-δ function. However, the catalytic activity of tyrosine-phosphorylated PKC-δ is variably described as reducing, increasing, or even changing in terms of substrate specificity and cofactor requirements, and information on PKC-δ tyrosine phosphate cannot be extracted from the literature (59, 60, 71, 72). Considering that PKC-δ is a tyrosine phosphorylated by TPA (59), it induces CHO cell division and arrest (40). Tyrosine phosphorylation of PKC-δ may be related to cell proliferation and differentiation.

In addition, ATP activates PKC-δ through G protein-coupled receptors (73). Proteolytic reactions and ultraviolet radiation also activate PKC-δ by different mechanisms. In general, PKC-δ is activated by diacylglycerol after serine and threonine phosphorylation at the motif site, which induces enzyme activation and produces a catalytic fragment (36). PKC-δ activated by different mechanisms has different functions; thus, the role of PKC-δ in each signaling pathway needs to be further studied.

### Mechanism of PKC-Δ Involvement in Cardiovascular Disease

PKC-δ regulates myocardial contraction, plays a role in ischemic preconditioning, and participates in the pathogenesis of myocardial IRI and fibrosis.

#### Myocardial Ischemia/Reperfusion Injury

An important method for the treatment of acute MI is revascularization, i.e., myocardial I/R treatment. However, reperfusion therapy itself can cause myocardial damage. PKC-δ plays a key role in injury related to ischemic tissue reperfusion. At present, some controversies exist regarding the role of PKC-δ in the process of ischemia and reperfusion.

Some studies suggest that PKC-δ activity may promote reperfusion injury after ischemia (74), which is partly mediated by the translocation of PKC-δ to mitochondria, which results in reductions in ADP-linked mitochondrial respiration (75), tricarboxylic acid cycle activity (76), cell pH, and ATP production rates (77). Mitochondria generate increased reactive oxygen species (75) and induce apoptosis by releasing cytochrome c (78). Inhibition of PKC-δ translocation with specific peptide inhibitors during reperfusion can block these effects and protect the heart from injury (75–78).

Inagaki et al. applied δv1-1 (a peptide inhibitor selective for PKC-δ) in an *in vivo* porcine model of AMI and showed that a PKC-δ inhibitor may improve the outcome of reperfusion in patients with AMI (77), Specifically, δv1-1 reduces the infarct area and troponin T levels in blood. Importantly, δv1-1 treatment improved the 30-min ejection fraction after reperfusion in pigs. The I/R-induced PKC-δ shift was completely inhibited by δv1-1 treatment in these hearts (41, 77, 79), indicating that I/R-induced myocardial injury was mediated by PKC-δ *in vivo*. Treatment with δV1-1 during *ex vivo* reperfusion can significantly improve functional recovery and improve myocardial ATP, phosphocreatine, and intracellular pH recovery during reperfusion (77). Therefore, δV1-1 may improve cardiac function recovery by reducing the time required to restore myocardial energy and pH during reperfusion (77). Studies have indicated that the use of PKC-δ selective inhibitors during IRI can reduce the injury of isolated perfused rat hearts (41, 80). Another study also noted that PKC-δ inhibitors can prevent replication *in vivo* in pig AMI models (77). In PKC-δ knockout mice, reductions in myocardial injury and infarct area were noted after coronary artery ischemia (41, 77, 81). The activation of PKC-δ in mitochondria contributes to I/R injury by decreasing intracellular pH and ATP production; increasing the accumulation of mitochondrial ROS; inhibiting the AKT-BAD phosphorylation pathway; promoting the release of cytochrome c, the activation of caspase-3, and the induction of apoptosis; and promoting I/R injury (76, 77, 82, 83). These results indicate that PKC-δ is an important component of the apoptosis signaling pathway. During myocardial I/R, phosphorylated PKC-δ translocates to mitochondria, and the release of cytochrome C increases significantly. These studies indicate that reperfusion injury following cardiac ischemia is mediated at least in part by the activation of PKC-δ. The inhibition of PKC-δ may reduce injury-related pathology in the process of reperfusion.

Rottlerin is a natural polyphenol compound that can inhibit PKC-δ. Studies have indicated that Ca^2+^-activated large conductance K^+^ (BKCa) activation can reduce severe ischemia induced by MI, promote survival, and improve heart and blood vessel function (84–89). Rottlerin can protect the heart by directly activating BKCa channels (90–92). Clements et al. demonstrated that rottlerin increases myocardial contraction and coronary perfusion by activating BKCa^++^ channels after cold cardiac arrest in isolated rats (93). Zheng et al. demonstrated that rottlerin significantly reduced apoptosis induced by reperfusion (94). Kai-9803 is a PKC-δ inhibitor; mechanistically, kai-9803 destroys the binding of PKC-δ and its activated C kinase receptor to prevent PKC-δ from mitochondrial localization during myocardial I/R (78). KAI-9803 reduces I/R injury in animal models with acute MI (77). Research indicates that it is necessary to further test kai-9803 as an adjuvant therapy for ST segment elevation MI (74). The I/R-induced PKC-δ shift (50% greater than the base) was completely inhibited by δv1-1 treatment in these hearts (41, 77, 79), indicating that I/R-induced myocardial injury was mediated by PKC-δ *in vivo*. These results suggest that inhibition of PKC-δ translocation inhibits apoptosis induced by reperfusion injury.

Previous reports have shown that apoptosis is a component of cardiomyocyte death during reperfusion (95). Myocardial I/R injury can activate endoplasmic reticulum (ER) stress. Continuous ER stress with a subsequent increase in intracellular Ca^2+^ concentration leads to apoptosis. *In vivo*, CaSR can activate PKC-δ to induce cardiomyocyte apoptosis through the ER stress-related apoptosis pathway (96). After IRI, the transformation of Caspase-3 into the active cleaved form increased significantly (97). However, the activity of Caspase-3 was greatly reduced by δ v1-1 treatment. These results suggest that inhibition of PKC-δ translocation can inhibit apoptosis induced by reperfusion injury. Murriel et al. demonstrated that PKC-δ activity and mitochondrial translocation at the beginning of reperfusion can promote the accumulation and dephosphorylation of apoptotic bad (Bcl-2-related death promoter), dephosphorylation of Akt, release of cytochrome c, cleavage of PARP (ADP ribose) polymerase, and DNA labeling (78). This finding suggests that PKC-δ activation plays a key role in promoting apoptosis in the cardiac response after ischemia and reperfusion. The application of PKC-δ inhibitors can inhibit this process and significantly reduce PKC-δ-induced apoptosis during reperfusion.

However, another study noted that PKC-δ knockout does not protect against but exaggerates I/R injury after pretreatment; in addition, the PKC-δ gene knockout-treated group had a larger infarct area than the untreated group (98). Mayr et al. provided evidence for loss of preconditioning-induced cardio protection in PKC-δ-deficient mice (99). In a related study, Fryer et al. used rottlerin, a PKC-δ selective antagonist, to observe its inhibitory effect on IPC-induced myocardial protection 30 min after ischemia and 2 h before reperfusion, suggesting that PKC-δ does not play an important role in IPC (100). A randomized controlled trial suggests that selective inhibition of PKC-δ does not reduce the biomarker of myocardial injury during PCI in patients with acute ST segment elevation MI (101).

Three recent studies suggest that PKC-δ may play a role in I/R through different signaling pathways. Remote ischemia per conditioning (RPerC) exhibits cardiac protective effects, and a study by Zhang et al. suggested that the underlying mechanism may occur through activation of opioid receptors and the NO-PKC-KATP channel signaling pathways (102). Diabetic myocardium shows higher vulnerability after IRI). It has been demonstrated that glucagon-like peptide-1 (GLP-1) has a protective effect on cardiomyocytes. High glucose-induced overexpression of PKC-δ in H9c2 cells destroys the GLP-1 receptor anti-apoptosis signaling pathway by inhibiting Akt phosphorylation (103). PKC-δ knockdown significantly restored the cardioprotective effects of GLP-1 in high glucose-cultured H9c2 cells (103). This finding suggests that high glucose-induced PKC-δ overexpression can reduce the protective effect of GLP-1 on the heart by downregulating GLP-1R and inhibiting the antiapoptotic signaling pathway of the GLP-1 receptor (103). Changes in mitochondrial metabolism are the primary cause of cardiovascular disease, and succinate is the mediator of mitochondrial dysfunction. The results showed that extracellular succinic acid promoted the transport of dynamic protein-related protein-1 (drp1) to mitochondria through the activation of PKC-δ. In addition, activation of extracellular signal regulated kinase-1/2 (ERK1/2) induced Gpr91-dependent phosphorylation of mitogen factor (MFF), leading to mitochondrial dysfunction and cardiomyocyte apoptosis. In the process of myocardial ischemia, succinic acid release activated Gpr91, which induced mitosis by regulating PKC-δ and ERK1/2 signaling branches. These findings indicate that inhibition of extracellular succinic acid mediated Gpr91 activation may represent a potential therapeutic strategy to protect myocardial cells from ischemia injury.

The role of PKC-δ in I/R may be affected by numerous factors. PKC-δ inhibitor content is related to the role of PKC-δ in I/R. Lower δV1 expression levels can inhibit ischemic-stimulated PKC-δ translocation, thereby protecting the heart from IRI. However, the severity of cardiomyopathy increases when δV1 is gradually expressed at higher levels in the heart (104). In the pretreated heart before ischemia, the ratio of proapoptotic PKC-δ and prosurvival PKC ε in mitochondria is regulated to determine the final fate of cells (83). In addition, different signal transduction pathways may also play an important role.

#### Fibrosis

Fibrosis can occur in a variety of organs. The main pathological changes are increased fibrous connective tissue and reduced parenchymal cells in organ tissues. Continuous progression of fibrosis can cause organ structure damage and functional decline and even failure, which seriously threatens human health and life. Fibrosis can lead to myocardial malfunction. Cardiac fibrosis occurs in a variety of cardiac pathological conditions. Cardiac fibrosis can reduce contractility and ultimately lead to heart failure. In addition, the period of CF will increase the production of extracellular matrix, destroy the myocardial circuits, induce arrhythmia, and even cause sudden cardiac death (105). Protein kinase C has been shown to modulate specific events leading to collagen deposition (106). The PKC-δ selective peptide inhibitor δV1-1 increased TGF-β1-induced proliferation (106). The data reported by Braun and Mochly-Rosen provide evidence that PKC-δ inhibits neonatal rat cardiac fibroblast proliferation (107).

Collagen is the main component of the extracellular matrix. The regulation of extracellular matrix remodeling is primarily mediated by cardiac fibroblasts (108–110). Under disease conditions, increased accumulation of interstitial collagens leads to fibrosis (111). Proliferation plays a key role in cardiac fibrosis. Ang II is an effective activator of CF, and activation of Ang II type 1 receptors can stimulate cell proliferation and *de novo* collagen synthesis (112, 113). Recent studies show that PKC-δ has a role in Ang II-induced cardiac fibrosis (114). Myofibroblasts (myoFb) represent the largest number of fibroblasts present in myocardial tissue. These cells can synthesize and maintain extracellular matrix, have autocrine and paracrine functions, and play an important role in maintaining normal heart function. Previous studies have demonstrated that Ang II induces myoFb proliferation and that myoFb is associated with cardiac fibrosis (115–119). When myocardial damage occurs for various reasons, fibroblasts proliferate, transform into myofibroblasts, increase extracellular matrix deposition, and cause cardiac fibrosis. PKC-δ-overexpressing myoFb showed a significant increase in PKC-δ activity and collagen expression. In addition, treatment with rottlerin, a PKC-δ inhibitor, significantly abrogated PKC-δ activity and Ang II-induced collagen expression in myoFb (114). A study by Olson et al. suggested that PKC-δ participates in transactivation of Ang II-induced cardiac fibroblasts ERK1/2 (a member of the tyrosine kinase pathway) (120). Chintalgattu and Katwa suggested that Ang II mediates the activity of PKC-δ and that inhibiting PKC-δ can abrogate Ang II-induced kinase activity. PKC-δ plays a key role in Ang II-induced collagen expression/fibrosis (114). Connective tissue growth factor (CTGF) is believed to play a variety of biological roles, including regulation of fibroblast proliferation, collagen synthesis, and apoptosis (121). Ang II increases CTGF expression *in vivo* (122). He et al. directly tested the regulation of CTGF expression by PKC-δ isoform activation after Ang II stimulation *in vitro* and *in vivo*, and the results indicated that PKC-δ inhibition may aggravate the expression of CTGF and potential fibrosis (123). Inhibition of PKC-δ leads to strong induction of CTGF expression after Ang II treatment (123).

## PKC-δ in Cardiovascular Disease

PKC-δ is involved in many cardiovascular diseases, such as atherosclerosis, hypertension, myocardial hypertrophy, and heart failure (Table 2). Our current concepts regarding the cardiac actions of PKC-δ are derived in large part from studies that rely on peptide translocation inhibitors or studies that characterize the phenotype of nPKC knockout mice (140).

**Table 2:** The mechanism of PKC-δ in different cardiovascular diseases.

**Diseases**	**Biomolecules**	**Function**	**References**
Atherosclerosis	Macrophages	Promote lipid accumulation and foam cell formation	(124)
	TM	Mediates macrophage differentiation	(125)
	Monocytes/Macrophages	Decrease the number of monocytes and inflammatory responses	(125)
	Ox-LDL	Induces endothelial cell apoptosis	(126)
	VSMCs	Inhibits intimal hyperplasia after arterial injury	(49)
Myocardial hypertrophy	FAK-S910	MEK5 and ERK5 mediate FAK-S910 phosphorylation, PKC-δ regulates upstream MEK5-ERK5	(127)
	Ventricular myocytes	Induce ventricular muscle apoptosis	(128)
	Hypertrophic myocytes	Regulate Ang II-induced apoptosis of hypertrophic cardiomyocytes	(129)
Heart failure	Mitochondria	Reduced the biological functions of mitochondria	(130)
	Protein kinase D	Influence HDAC5- and/or CREB-dependent transcriptional programs that promote cardiomyocyte growth/survival and induce cardio protection	(99, 131–133)
	IL-6	Promote the secretion of IL-6 induced by isoproterenol, thereby mediating poor myocardial remodeling.	(134, 135)
	Cardiomyocytes	Downregulate sarcoplasmic reticulum Ca^2+^ ATPase (SERCA2) gene expression and reduce myocardial contractility	(136)
	Hypertrophic cardiomyocytes	Regulates myocardial cell diastolic function, induces apoptosis and is associated with myocardial cell necrosis	(129)
Hypertension	VSMCs	Decreased sensitivity of vascular smooth muscle to EDRF/no	(137, 138)
	IH	Enhance vasoconstriction	(139)

*TM, thrombomodulin; VSMCs, vascular smooth muscle cell; FAK, focal adhesion kinase; IH, intermittent hypoxia*.

### Atherosclerosis

Atherosclerosis is a chronic inflammatory reaction and the most common pathological cause of cardiovascular disease. The development of atherosclerosis involves many cells (including endothelial cells, VSMCs, macrophages, and others) (141). Macrophages participate in the intracellular accumulation of lipids and the formation of foam cells in early atherosclerotic lesions (124). Macrophage uptake of oxidized lipoprotein particles (ox-LDL) and foam cells is the first step in the development of atherosclerosis (142). Studies have shown that macrophage differentiation is an important process in the development of atherosclerosis, and thrombomodulin (TM), a transmembrane glycoprotein expressed on the cell surface, is a signaling molecule that mediates macrophage differentiation (125). Tsai et al. provided a new perspective on the role of TM in the differentiation of macrophages during the development of atherosclerosis and found that PKC-δ is highly expressed in human atherosclerotic arteries and infiltrates with TM in CD68-positive macrophages. TM-PKC-δ interactions in macrophages may contribute to the formation of atherosclerosis (125). Activation of monocytes/macrophages contributes to atherosclerosis, and the activation of the PKC-δ isoform in monocytes can decrease the number of monocytes and inflammatory responses in the arterial wall, thus slowing the development of atherosclerosis (143). Curcumin inhibits matrix invasion by inhibiting the PKC-δ/NADPH oxidase/ROS signaling pathway during monocyte macrophage differentiation to intervene in the process of atherosclerosis (144). PKC-δ may be closely related to the occurrence of atherosclerosis (144). Under pathological conditions, endothelial cell apoptosis is the initial step in atherosclerosis and contributes to the subsequent atherosclerosis process (145). Ox-LDL (an important atherosclerotic factor) induces endothelial cell apoptosis as an important process in the development of atherosclerosis. LOX-1 is overexpressed in atherosclerotic endothelial cells (146). Zhou and Chen's research provides evidence that lincRNA-p21 is the main mediator of human vascular endothelial cell apoptosis and that LOX-1 expression is induced by ox-LDL and works by activating PKC-δ (126). Smooth muscle cell (SMC) accumulation is a key event in the development of atherosclerosis. A study of a rat arterial injury model found that PKC-δ upregulation is accompanied by the apoptosis of vascular wall VSMCs and that PKC-δ inhibits intimal hyperplasia after arterial injury by stimulating apoptosis of VSMCs (49). Leitges et al. isografted vein segments from PKC-δ^−/−^ mice and found more severe atherosclerosis compared with that noted in PKC-δ^+/+^ mouse vein grafts, which correlated with decreased SMC lesions (43). Above evidence indicated that PKC-δ activation enhances the apoptosis of SMCs and prevents the accumulation of SMCs, which is of great significance in the amelioration of atherosclerosis.

### Myocardial Hypertrophy

PKC-δ is found in adult rat cardiac myocytes and cultured neonatal ventricular myocytes (34). PKC-δ is also detected in total protein extracts from isolated adult ventricular myocytes (147). PKC-δ has been implicated in the downregulation of SERCA2 [sarco(endo)plasmic reticulum Ca2^+^-ATPase] expression, a characteristic feature of the hypertrophic phenotype. A study of a pressure overload model of abdominal aortic coarctation showed that PKC-δ self-phosphorylation and activity increased in the process of ventricular hypertrophy, and PKC-δ expression also increased significantly with the gradual emergence and aggravation of left ventricular hypertrophy (148). The pressure overload model of ascending aortic stenosis also confirmed that PKC-δ expression increased during left ventricular hypertrophy (149). The Dahl salt-sensitive rat heart failure model also confirmed that PKC-δ expression continued to increase during the transformation from cardiac hypertrophy to heart failure. In the volume overload model, PKC-δ protein and mRNA levels also increased significantly (150). Yi et al. suggested that FAK may represent a candidate gene for nuclear hypertrophy gene transcription and RNA processing in chronic hypertensive cardiac hypertrophy (151). FAK-S910 phosphorylation is necessary for the formation of new costumeries in the process of myocardial cell remodeling. FAK-S910 phosphorylation is partially mediated by MEK5 and ERK5, and PKC-δ regulates upstream MEK5-ERK5 (127). Rottlerin treatment or PKC-δ-KD (kinase dead PKC-δ) expression inhibits PKC-δ, thereby inhibiting apoptosis induced by a variety of stimuli (152, 153). Adenovirus transfection studies have also shown that activation of PKC-δ in ventricular myocytes can directly induce ventricular muscle apoptosis (128). In summary, PKC-δ regulates myocardial cell diastolic function, induces apoptosis and is associated with myocardial cell necrosis. Apoptosis of hypertrophic cardiomyocytes is the key mechanism of heart failure. Cardiac hypertrophy is an adaptive process to increase hemodynamic overload. Rottlerin reduces the apoptotic rates induced by Ang II in mildly hypertrophic myocytes and in moderately hypertrophic myocytes, but rottlerin did not affect the apoptotic rate induced by Ang II in serum-free cultured myocytes. These results suggest that inhibition of PKC-δ reduces Ang II-induced apoptosis of hypertrophic cardiomyocytes and that PKC-δ is possibly involved in the apoptotic process of hypertrophic cardiomyocytes (129).

### Heart Failure

As a stress protein, PKC-δ is activated in the early stage of pressure overload and heart failure (154). Yang et al. concluded that cardiac ROS-dependent activated PKC-δ colocalizes with mitochondria, and Drp1-dependent mitochondrial fission and fragmentation reduced the biological functions of mitochondria, resulting in cardiac failure (130). Myocardial ischemia and MI are the two most common reasons for heart failure (155). The degree of cardiac dysfunction after I/R injury reflects the level of myocyte injury and apoptosis (156). Although the role of PKC-δ in the pathogenesis of heart failure is not conclusive, many studies have confirmed that PKC-δ is associated with the occurrence of heart failure. Shende et al. reported that PKCβII deficiency and increased PKC-δ are important reasons for rector-deficient heart dysfunction after pressure overload (157). PKC-δ act as a lipid-independent enzyme in the soluble fraction of cardiomyocytes undergoing oxidative stress (57, 158). In addition, PKC-δ can contribute to receptor-dependent pathways that activate protein kinase D and thereby influence HDAC5- and/or CREB-dependent transcriptional programs that promote cardiomyocyte growth/survival and induce cardio protection (99, 131–133). IL-6 is a multifunctional cytokine that is an important biomarker that predicts the severity, prognosis, and mortality of heart failure (159). IL-6 is involved in mediating adverse myocardial remodeling (134). PKC-δ mediates IL-6 secretion induced by isoprenaline (135).

Compensatory cardiac hypertrophy caused by hypertrophic cardiomyopathy is the most important factor leading to heart failure. PKC-δ regulates myocardial cell diastolic function, induces apoptosis, and is associated with myocardial cell necrosis (129). Apoptosis of hypertrophic cardiomyocytes is the key mechanism of heart failure. Myocardial hypertrophy is a process that adapts to the increase in hemodynamic load. Rottlerin is a specific inhibitor of PKC-δ that can inhibit the apoptosis rate of mildly hypertrophic cardiomyocytes and moderately hypertrophic cardiomyocytes (129). These results suggest that inhibition of PKC-δ reduces Ang II-induced apoptosis of hypertrophic cardiomyocytes (129). Low-dose PKC-δ is beneficial to the adaptation of cardiomyocytes to exercise stimulation (160), This mechanism may protect the heart muscle by regulating the mitochondrial K^+^ channel (161). Long-term PKC-δ expression can damage cardiomyocytes, and PKC-δ can significantly downregulate sarcoplasmic reticulum Ca^2+^ ATPase (SERCA2) gene expression, and reduce myocardial contractility (136).

PKC-δ is related to cardiomyocyte hypertrophy, which can move from cytoplasm to mitochondria, and then trigger apoptosis signal pathway. Xie et al. shows that the mitochondrial pathway of PKC-δ determines the apoptosis signal of hypertrophic cardiomyocytes (162). PKC-δ may be involved in the transition from cardiac hypertrophy to apoptosis (129). In a dog model of pacing-induced heart failure, the apoptosis rate increased significantly (78, 163), and apoptosis was further increased in heart failure (164). In addition, Ozgen et al. found that the Galpha(q)- PKC-δ-PKD-CREB-Ser (126) phosphorylation pathway was involved in cardiac remodeling and could be used as a target for heart failure treatment (132).

### Hypertension

A high level of PKC activity is a typical characteristic of different types of arterial hypertension (137, 165). The main reason for the reduced aortic endothelium-dependent diastolic function in spontaneously hypertensive rats is the decreased sensitivity of vascular smooth muscle to EDRF/no, which is caused by the increase in PKC activity (137, 138). Novokhatska et al. showed that increasing the activity of the PKC-δ isoform in the vascular wall can decrease the endothelium-dependent relaxation induced by acetylcholine, which is related to the tall outward potassium current of spontaneously hypertensive rats. In addition, an RNA interference technique can be used to silence the PKC-δ isoform gene to restore normal vascular contractility in hypertensive rats (166).

Phosphorylation of tyrosine residues in focal adhesion kinase (FAK) is necessary for cardiomyocytes to respond to growth factor and mechanical load M, and serine and tyrosine phosphorylation of FAK is significantly increased in rats with hypertension (151). Related reports showed that the phosphorylation level of serine residues in FAK increased significantly in hypertensive rats, and different phosphorylation sites can regulate the late subcellular localization of FAK (167). Gerasymenko et al. showed that transgenic lettuce plants producing shRNA targeting PKC-δ can be used as a raw material for the development of novel drugs for hypertensive treatment (168).

Obstructive sleep apnea (OSA) can cause intermittent hypoxia (IH), which is one of the risk factors for hypertension (169). Studies in rodent models have shown that prolonged sleep apnea can lead to vascular dysfunction (170). Kanagy et al. indicated that IH exposure increases plasma ET-1, which may contribute to hypertension (171). Given the increase in circulating ET-1 levels, rats exposed to IH showed an increased contractile response to ET-1 in the mesenteric artery (172). The enhancement of vasoconstriction induced by IH is mediated by PKC-δ, and ET-1 increases the level of self-phosphorylation of PKC-δ (139). In addition, rottlerin reduces ET-1-mediated contraction in the arteries of rats exposed to IH (169). These results indicate that PKC-δ is involved in the hypertension response to ET-1 after IH exposure.

## Discussion

PKC-δ is a serine/threonine kinase that plays a key role in growth regulation and tissue remodeling. PKC-δ activation mediates the pathological process of various cardiovascular diseases. Traditional PKC-δ activation models focus on the anchoring of physiological second messengers (such as DAG diacylglycerol) or tumor-promoting PSs (such as PMA) in the active conformation of PKC membranes. However, this receptor-driven, lipid cofactor-dependent PKC activation mechanism involving membrane-associated anchoring proteins does not adequately explain PKC-dependent phosphorylation of no membranous proteins. Therefore, traditional PKC-δ activation models must be broadened to allow for stimulation-specific differences in PKC-δ signals during growth factor stimulation and oxidative stress. New activation methods, such as tyrosine phosphorylation, provide the possibility for further research on PKC-δ. Many studies have relied on *in vitro* kinase analysis to address tyrosine phosphorylation-dependent changes in PKC-δ function. However, the catalytic activity of tyrosine-phosphorylated PKC-δ is variably described as reducing, increasing, or even changing in terms of substrate specificity and cofactor requirements. At present, there is no uniform conclusion on PKC-δ tyrosine phosphorylation. Further research is needed. PKC-δ activation is achieved by coordinating phosphorylation and transport events. When evaluating PKC-δ signaling pathways, these two mechanisms must be considered. In addition, a drug inhibitor should be designed to eliminate the effects of PKC-δ.

PKC-δ can also be used as the target of SRC and RAS transformation or the phosphorylation of tyrosine residues stimulated by PMA, epidermal growth factor, platelet-derived growth factor, and hydrogen peroxide. However, the precise biological results of PKC-δ tyrosine phosphorylation have been difficult to decipher due to the presence of the regulatory domain (tyr52, tyr155, and tyr187), catalytic domain (tyr512 and tyr523), and hinge region (tyr311 and tyr332) in PKC-δ. However, exclusively focusing on the driving effect of PKC-δ phosphorylation may be myopic (or even misplaced). In view of recent evidence, PKC-δ phosphotyrosine can also be used as the docking site of other signal proteins. The role of PKC-δ as a signal-regulated scaffold can be the basis of some of its effector functions.

PKC-δ activation signaling regulates myocardial contraction and plays an important regulatory role in myocardial I/R injury and cardiac fibrosis. At present, research on the role of PKC-δ in I/R injury is mainly based on an *in vivo* AMI pig model, an *in vitro* rat heart model, and a PKC-δ gene knockout mouse model. At present, some controversies about the role of PKC-δ in the process of ischemia and reperfusion exist. Evidence in mouse and pig models suggest that PKC-δ promotes I/R injury, and inhibition of PKC-δ can block these effects. Inhibiting PKC-δ translocation with specific peptide inhibitors during reperfusion can block these effects and protect the heart from harm (75–78). The study of PKC-δ inhibitors *in vivo* and *in vitro* suggests that inhibition of PKC-δ plays a role in reducing the infarct area and troponin T level; inhibiting the I/R-induced PKC-δ shift; improving functional recovery during reperfusion; and improving myocardial ATP, creatine phosphate, and intracellular pH recovery. In addition, inhibition of PKC-δ reduces caspase-3 activity and has a positive role in inhibiting apoptosis.

These results suggest that inhibition of PKC-δ may be an important target for drug development to prevent irreversible cardiac injury during reperfusion. However, a random controlled trial (101) suggested that selective inhibition of PKC-δ does not reduce the biomarkers of myocardial injury in PCI of acute ST segment elevation MI, underscoring the differences in animal models and humans. Further clinical trials are necessary to verify the current conclusions and then determine future research directions. The role of PKC-δ in I/R may be related to the amount of PKC-δ inhibitor. A study (104) indicated that lower δV1 expression levels can inhibit ischemic-stimulated PKC-δ translocation, thereby protecting the heart from IRI. However, the severity of cardiomyopathy increases when δV1 is gradually expressed at higher levels in the heart. In addition, recent studies have found that PKC-δ may play a role in I/R through specific signaling pathways, which provides a new idea for the study of the mechanism of PKC-δ in I/R.

At present, studies have shown that PKC-δ is related to the occurrence of heart failure, and its mechanism may involve hypertrophic hearts. We should explore the mechanism of PKC-δ and provide new ideas for the prevention and treatment of heart failure. PKC-δ was highly expressed in human atherosclerotic arteries infiltrated with TM and macrophages positive for CD68, suggesting that the interaction of macrophage tm-PKC-δ may participate in the formation of atherosclerosis (125). The activation of monocytes/macrophages contributes to the formation of atherosclerosis. Activation of the PKC-δ subtype of monocytes can reduce the number of PKC-δ subtypes and the inflammatory response of the arterial wall, thus slowing the development of atherosclerosis (143). Curcumin inhibits matrix invasion during monocyte phage differentiation by inhibiting the PKC-δ/NADPH oxidase/ROS signaling pathway, thus interfering with atherosclerosis (144). Zhou and Chen showed that lincrna-p21 was the main mediator of oxidized LDL-induced apoptosis and LOX-1 expression in human vascular endothelial cells by activating PKC-δ (126). PKC-δ upregulation was accompanied by VSMC apoptosis, and PKC-δ inhibited intimal hyperplasia by stimulating vascular smooth muscle cell apoptosis (49). At present, there is no unified theory regarding the mechanism of PKC-δ in atherosclerosis. Research on specific PKC-δ-targeting drugs should be performed in the future. The pressure overload model of abdominal aortic coarctation (148), the pressure overload model of ascending aortic stenosis (149), the Dahl salt-sensitive rat heart failure model and the volume overload model have been studied (150). Studies using these models confirmed that PKC-δ participates in cardiac hypertrophy. However, literature to support the specific mechanism is lacking. PKC-δ is involved in endothelium-dependent systolic and diastolic function in spontaneously hypertensive rats and in the response of hypertension to ET-1.

The role and mechanisms of PKC-δ in CVD are complex and involve different cardiovascular biological processes by mediating different biological factors. PKC-δ mediates lipid accumulation and foam cell formation, macrophage differentiation, monocyte number, inflammatory response, and endothelial cell apoptosis through the regulation of macrophage (124), TM (125), monocyte/macrophage (125), OX-LDL (126), VSMCs (49), and other biological factors. However, its role in heart failure is mainly to regulate cardiac remodeling and myocardial contractility by regulating IL-6 secretion (134), SERCA2 gene expression (136), and diastolic function of cardiomyocytes. PKC-δ regulates hypertension by mediating VSMCs (137, 138) and IH (139), reduces the sensitivity of vascular smooth muscle to EDRF/NO, and enhances vasoconstriction.

Protein kinase C inhibitors and activators are used for the diagnosis and treatment of various PKC-associated diseases, such as cancers, neurological diseases, cardiovascular diseases, and infections. However, the application of PKC in cardiovascular diseases still needs exploration. Flosequinan is a drug that can generally inhibit PKC isoenzymes. Studies have found that it produces continuous hemodynamic benefits in heart failure (173). PKC-ε activators such as volatile, anesthetics, and adenosine acadesine are used in the treatment of coronary artery bypass grafting and acute MI (174–177). Lincoff et al. concluded that selective inhibition of PKC-δ with intravenous infusion of delcasertib during PCI for acute STEMI in a population of patients treated according to the contemporary standard of care did not reduce biomarkers of myocardial injury (101). At present, there are relatively few clinical trials of cardiovascular diseases that target PKC, and the results are not as satisfactory as the results of animal experiments. The high homology of PKC isoenzymes makes the clinical transformation of PKC challenging, and drugs targeting a single isoenzyme are difficult to achieve. In addition, unpredictable adverse effects, insufficient therapeutic effects, insufficient preclinical studies, and lack of blood biomarkers are also important reasons leading to unsatisfactory clinical results.

This article reviews the role of PKC-δ in the development of cardiovascular disease. In recent years, an increasing number of studies have found that PKC-δ plays an important role in the occurrence, development, and prevention of cardiovascular diseases. The discussion of its activation mechanism has expanded from traditional activation methods, such as diacylglycerols and PSs, to tyrosine phosphorylation. Disease research has also been extended to hypertrophic cardiomyopathy, atherosclerosis, hypertension, and heart failure. In-depth drug research on PKC-δ is also increasing, and the role of PKC-δ in the pathogenesis of CVD is currently being explored through drug research. The role of PKC-δ as a potential molecular target for cardiovascular intervention requires further research. This review article provides an important theoretical basis to better understand current progress in research on PKC-δ in cardiovascular disease.

## Perspective

Although PKC-δ is a very promising target for cardiovascular diseases, there are still many limitations to be solved urgently. First, despite the role of PKC-δ has been proved in previous experiments, the results are not satisfying in pre-clinical trials. This may partly be explained by the difference of animal models and corresponding human diseases. Second, there is a high degree of substantial homology between PKC isoenzymes. Many PKC isoenzymes exist in the same cell and are activated by the same stimulus. This feature of high homology makes it difficult to develop a single pharmacological tool that affects PKC-δ. Post-translational modifications of PKC-δ and upstream molecular regulation of the PKC-δ activation pathway may be promising targets for drug discovery. Third, there are few studies concerning the role of PKC-δ in specific heart diseases, such as alcoholic cardiomyopathy and obese cardiomyopathy currently, which suggests further research are necessary. Fourth, PKC-δ is widely expressed in various tissues and cells throughout the body and lacks specificity. In the future, the development of inhibitors or activators for PKC-δ subtypes may provide a way to tackle this difficulty. In addition, PKC-δ-related blood biomarkers are insufficient. To date, there are no PKC specific biomarkers to indicate dose adequacy or predict phenotypic outcomes, which can also be improved in future studies.

## Conclusion

The current evidence proves that the activation and inhibition of PKC-δ has an important regulatory role in CVD. PKC-δ-mediated apoptosis, inflammatory, cardiac remodeling, and vascular contraction and dilatation lead to several biology effects, and thus bring hope for the treatment of CVD. However, there are still many limitations in current research, more in-depth research can be further conducted to explore new biomarkers and the specific functions of different PKC-δ subtypes.

## Author Contributions

MG and D-ZS: conceptualization and supervision. L-nM and DP: literature search and summary. J-pD: project administration. L-nM and P-fC: writing original draft. MG, D-ZS, and JG: writing review and editing. D-ZS, JS, and Y-qY revised the manuscript. All authors contributed to the article and approved the submitted version.

## Funding

This work was supported by National Natural Science Foundation of China (Grant no. 81904025), Fundamental Research Funds for the Central Public Welfare Research Institutes (Grant no. ZZ13-YQ-016 and ZZ13-YQ-016-C1), and National Natural Science Foundation of China Youth Fund Project (Grant no. 81904046).

## Conflict of Interest

The authors declare that the research was conducted in the absence of any commercial or financial relationships that could be construed as a potential conflict of interest.

## Publisher's Note

All claims expressed in this article are solely those of the authors and do not necessarily represent those of their affiliated organizations, or those of the publisher, the editors and the reviewers. Any product that may be evaluated in this article, or claim that may be made by its manufacturer, is not guaranteed or endorsed by the publisher.
